# Dapagliflozin Treatment Augments Bioactive Phosphatidylethanolamine Concentrations in Kidney Cortex Membrane Fractions of Hypertensive Diabetic db/db Mice and Alters the Density of Lipid Rafts in Mouse Proximal Tubule Cells

**DOI:** 10.3390/ijms24021408

**Published:** 2023-01-11

**Authors:** Mohammed F. Gholam, Lauren P. Liu, Louis A. Searcy, Nancy D. Denslow, Abdel A. Alli

**Affiliations:** 1 Department of Physiology and Aging, University of Florida College of Medicine, Gainesville, FL 32610, USA; 2 Department of Basic Medical Sciences, College of Medicine, King Saud bin Abdulaziz University for Health Sciences, Jeddah 22384, Saudi Arabia; 3 Center for Environmental and Human Toxicology, Department of Physiological Sciences, College of Veterinary Medicine, University of Florida, Gainesville, FL 32611, USA; 4 Department of Medicine Division of Nephrology, Hypertension and Renal Transplantation, University of Florida College of Medicine, Gainesville, FL 32610, USA

**Keywords:** diabetes, kidney, lipidomics, lipid rafts, dapagliflozin

## Abstract

In addition to inhibiting renal glucose reabsorption and allowing for glucose excretion, the sodium/glucose cotransporter 2 (SGLT2) inhibitor dapagliflozin may be efficacious in treating various comorbidities associated with type 2 diabetes mellitus (T2DM). The molecular mechanisms by which dapagliflozin exerts its beneficial effects are largely unknown. We hypothesized dapagliflozin treatment in the diabetic kidney alters plasma membrane lipid composition, suppresses extracellular vesicle (EV) release from kidney cells, and disrupts lipid rafts in proximal tubule cells. In order to test this hypothesis, we treated diabetic db/db mice with dapagliflozin (N = 8) or vehicle (N = 8) and performed mass spectrometry-based lipidomics to investigate changes in the concentrations of membrane lipids in the kidney cortex. In addition, we isolated urinary EVs (uEVs) from urine samples collected during the active phase and the inactive phase of the mice and then probed for changes in membrane proteins enriched in the EVs. Multiple triacylglycerols (TAGs) were enriched in the kidney cortex membrane fractions of vehicle-treated diabetic db/db mice, while the levels of multiple phosphatidylethanolamines were significantly higher in similar mice treated with dapagliflozin. EV concentration and size were lesser in the urine samples collected during the inactive phase of dapagliflozin-treated diabetic mice. In cultured mouse proximal tubule cells treated with dapagliflozin, the lipid raft protein caveolin-1 shifted from less dense fractions to more dense sucrose density gradient fractions. Taken together, these results suggest dapagliflozin may regulate lipid-mediated signal transduction in the diabetic kidney.

## 1. Introduction

Sodium–glucose co-transporters (SGLTs) are members of the solute carrier 5 family of glucose active transporters [1]. SGLT1 and SGLT2 subtypes are primarily responsible for the physiological transport of glucose in the kidney [1]. Almost 97% of the filtered glucose that is reabsorbed is carried out by the high-carrying capacity, low-affinity SGLT2 transporter, which is almost exclusively expressed in renal proximal tubular epithelial cells [2]. Mechanistically, SGLT2 inhibitors including dapagliflozin can reduce the maximum carrying capacity of tubular glucose absorption, which raises urine glucose excretion and lowers plasma glucose levels. In addition to their hypoglycemic actions, SGLT2 inhibitors have been demonstrated to offer blood pressure lower effects [3,4,5], weight-loss [6,7], and cardioprotective benefits [6,8,9] and to enhance glucose metabolism in epicardial fat [10]. Furthermore, dapagliflozin may improve lipoprotein profile and atherosclerosis development [11], in contrast to empagliflozin that raises low-density lipoprotein cholesterol levels [12]. The SGLT2 inhibitor empagliflozin has been shown to reduce levels of inflammatory factors including IL-1, IL-6, and IL-8 in models of diabetics [13] and decrease mitochondrial calcium overload and reactive oxygen species production in human endothelial cells, which are caused by high glucose levels [14]. A study by Chen et al. showed that dapagliflozin alleviated hyponatremia, hypokalemia, and hypochloremia in diabetic rats and came to the conclusion that this drug did not cause an electrolyte imbalance or significant volume depletion [15]. However, the exact mechanism for dapagliflozin is still largely unknown in terms of blood pressure regulation. Therefore, filling this critical knowledge gap could give us a better understanding of the mechanisms through which dapagliflozin regulates blood pressure, involving renal transporters.

The risk of cardiovascular disease, renal failure, and cerebrovascular events is increased by uncontrolled blood pressure, otherwise known as hypertension. Hypertension is a major public health concern [16]. Uncontrolled hypertension contributes to organ failure including renal failure [17]. It has been demonstrated that effective blood pressure management lowers the risk of stroke, heart attack, and heart failure [18]. Diuretics are one of the early treatment options for hypertension, but they can have considerable side effects that cause volume depletion and electrolyte imbalance, including hyponatremia and hypokalemia [19]. 

Several physiological and behavioral traits follow the 24 h cycle and are synchronized with the day/night cycle. In particular, the physiological processes that are related to the kidneys, including renal blood flow, glomerular filtration rate (GFR), blood pressure, renal cortico-medullary osmotic gradient, and tubular water and electrolyte transport, exhibit circadian rhythms and are thought to be regulated over a 24 h cycle, at least in part, by the intrinsic kidney clocks [20].

Extracellular vesicles (EVs), including exosomes, are specialized membranous vesicles that are released by all cells and found in bodily fluids including blood and urine [21]. They are more than just waste-exporting vesicles, as they enable the transfer of mRNAs, microRNAs, DNA, and proteins across cells [22]. EVs have been shown to allow for intercellular communication within the nephron [23] and play a role in signal transduction [24,25,26,27]. EVs are a rich source of biomarkers, and recent research has shown that they may play a role in the pathophysiology of hypertension [21]. 

The purpose of this study was to test our hypothesis that dapagliflozin treatment alters kidney cortex membrane lipids and the release of EVs into the urine of hypertensive diabetic db/db mice in a time-of-day-dependent manner. Additionally, we provide a discussion of potential beneficial effects of dapagliflozin to mitigate outcomes of salt-induced hypertension in the diabetic kidney at the level of changes in membrane lipids and EV release from renal epithelial cells. 

## 2. Results

### 2.1. Dapagliflozin Treatment Alters Membrane Lipid Composition in the Kidneys of Salt-Loaded Hypertensive Diabetic db/db Mice

**Figure 1:**
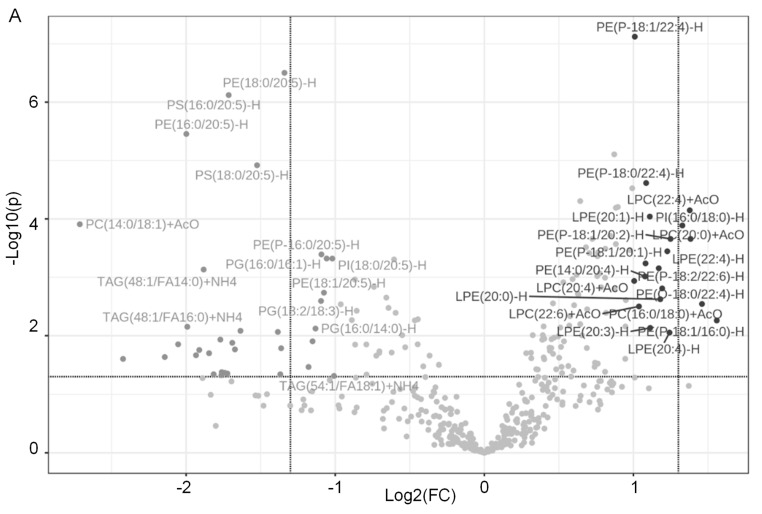

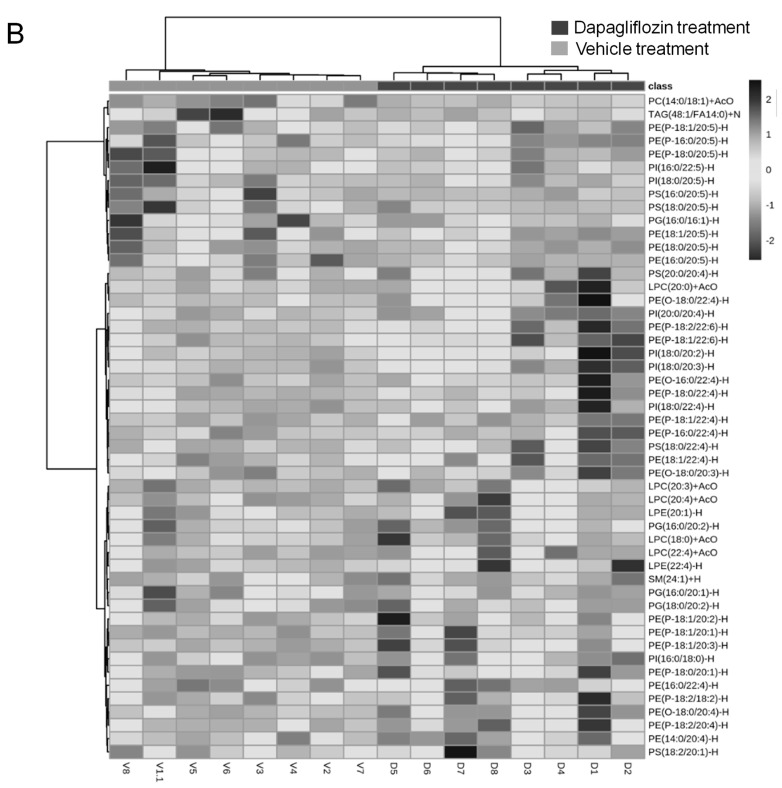
Analysis of lipids in the kidneys of dapagliflozin- or vehicle-treated salt-loaded hypertensive diabetic db/db mice. (**A**) Volcano plot showing 34 lipids that are significantly down-regulated and 19 lipids that are significantly upregulated in the kidneys of diabetic db/db mice treated with dapagliflozin compared to vehicle. A 2.0-fold change threshold and a *p*-value threshold of 0.05 were applied. Data were normalized by the mean and then to the median in MetaboAnalyst with Pareto scaling. The points representing specific lipids on the left (value < 0) are expressed at a lower level, and those on the right (value > 0) are expressed at a higher level, in the dapagliflozin group compared to the vehicle group. (**B**) Heatmap with Euclidean distance measure, Ward clustering method showing the top 50 lipids, with each colored cell on the map corresponding to a concentration value in the data table.

Diabetic db/db mice are known to develop hypertension upon salt loading [28]. Here, we salt-loaded male diabetic db/db mice for 10 days to induce hypertension and then treated the mice with either a vehicle or dapagliflozin for 14 days. The membrane lipids were extracted from the kidney cortex, and relative lipid concentrations were analyzed between the two groups. A total of 431 lipids were detected. A volcano plot shows several lipids are significantly downregulated, while other lipids are significantly upregulated in diabetic db/db mice treated with dapagliflozin compared to vehicle (Figure 1A). A heatmap shows the clustering of the top 50 lipids based on the relative lipid concentrations between the two treatment groups (Figure 1B). Triacylglycerols (TAGs), phosphatidylethanolamine (PEs), phosphatidylglycerols (PGs), phosphatidylserines (PSs), phosphatidylinositols (PIs), lysophosphatidylethanolamines (LPEs), and lysophosphatidylcholine (LPCs) are the main classes of lipids that were found to be differentially enriched in the membranes between dapagliflozin-treated and vehicle-treated diabetic db/db mice.

### 2.2. Decrease in TAGs in Kidney Cortex Membrane Fractions of db/db Mice Treated with Dapagliflozin

**Figure 2:**
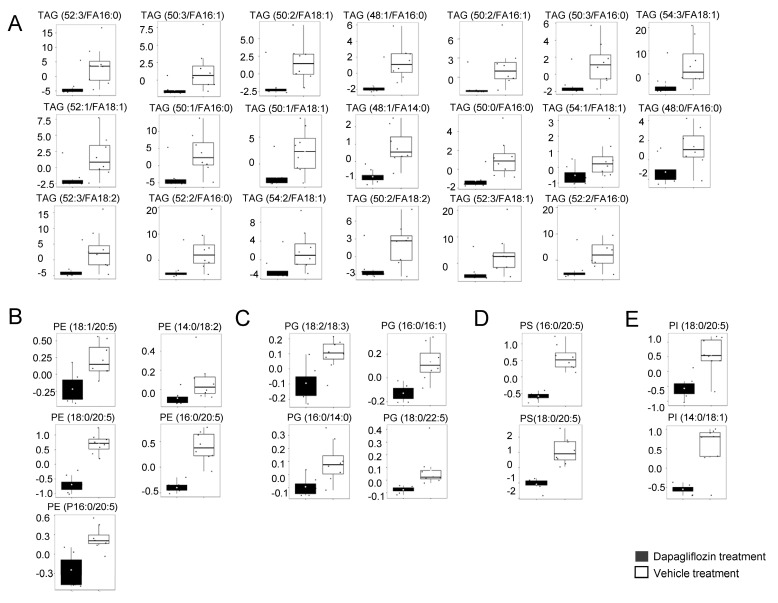
Lipids significantly downregulated in the kidneys of the dapagliflozin-treated salt-loaded hypertensive diabetic db/db mice compared to the vehicle-treated db/db mice. (**A**) Plots of triacylglycerols (TAGs), (**B**) plots of phosphatidylethanolamines (PEs), (**C**) plots of phosphatidylglycerols (PGs), (**D**) plots of phosphatidylserines (PSs), and (**E**) plots of phosphatidylinositols (PIs) significantly different between the two groups. The concentration of each lipid is given in µmol/g. The concentration of each lipid was normalized to the median. The black (left bar) represents the dapagliflozin group, and the white (right bar) represents the vehicle group.

A published study reported that the triacylglycerol (TAG) lipid architecture plays an important role in the progression of diabetic kidney disease [29]. Thus, one of the objectives of this study was to investigate whether the concentrations of TAGs are altered by dapagliflozin treatment. Our lipidomics data show several forms of TAGs are decreased in kidney cortex membrane fractions from diabetic db/db mice treated with dapagliflozin compared to the vehicle (Figure 2A). Similarly, there were lower amounts of various PEs, PGs, PS, and PIs in the membranes of dapagliflozin-treated diabetic db/db mice compared to vehicle-treated mice (Figure 2B–E). While there were multiple forms of several different lipid classes that were found to be reduced in concentration in the membranes of dapagliflozin treated mice, there were multiple forms of other lipid classes that were found to be increased in the membranes of the dapagliflozin group compared to the control group. 

### 2.3. Increase in LPCs in Kidney Cortex Membrane Fractions of db/db Mice Treated with Dapagliflozin

**Figure 3:**
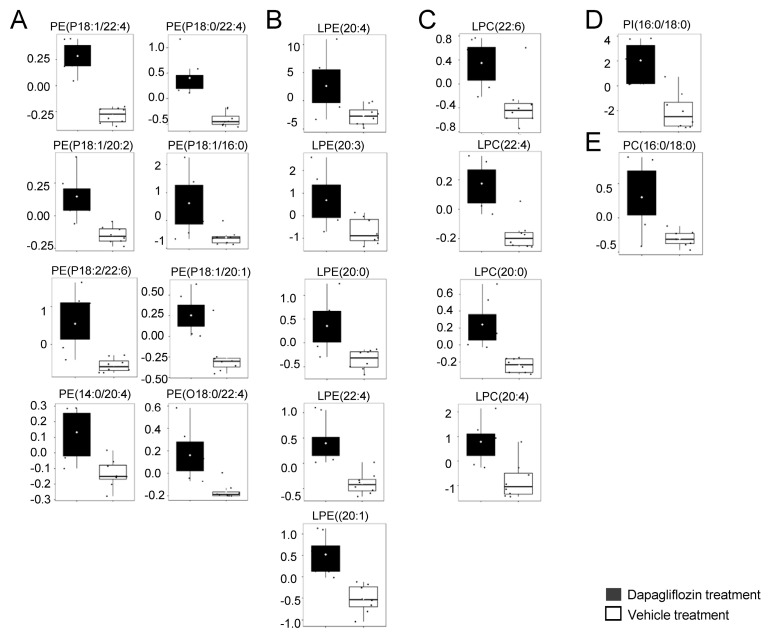
Lipids significantly upregulated in the kidneys of dapagliflozin-treated salt-loaded hypertensive diabetic db/db mice compared to the vehicle-treated db/db mice. (**A**) Plots of PEPs, PEOs, and PEs, (**B**) plots of lysophosphatidylethanolamines (LPEs), (**C**) plots of lysophosphatidylcholines (LPCs), (**D**) plots of phosphatidylinositols (PIs), and (**E**) plots of phosphatidylcholines (PCs). The concentration of each lipid is given in µmol/g. The concentration of each lipid was normalized to the median. The black (left bar) represents the dapagliflozin group, and the white (right bar) represents the vehicle group.

The accumulation of LPC 16:0 and 18:0 in the kidney and urine is associated with kidney function decline in patients with diabetic kidney disease [30]. Other studies have shown that PEs are important metabolites that promote the early onset of diabetic kidney disease [31]. These studies motivated us to investigate whether dapagliflozin treatment alters the concentration of various PCs and PEs in the kidneys of diabetic db/db mice. First, we show that LPC (22:6), LPC (22:4), LPC (20:0), and LPC (20:4) levels are higher in the kidney cortex membrane fractions of diabetic db/db mice treated with dapagliflozin compared to the vehicle (Figure 3). Other forms of LPCs including LPC 16:0 and 18:0 did not show a change between the two groups or were not detectable in each sample. While our data show various forms of PE are elevated in the membranes of kidneys from diabetic db/db mice treated with the vehicle (Figure 2B), there were several modified forms of PEs, in the form of PEPs and PEOs, that were elevated in dapagliflozin-treated mice compared to vehicle-treated mice (Figure 3A). Similar to these PEPs and PEOs, the concentrations of several forms of LPEs, LPCs, PI(16:0/18:0), and PC(16:0/18:0) were greater in the membranes of dapagliflozin-treated hypertensive diabetic db/db mice compared to the vehicle-treated mice (Figure 3B–E). Here, were show the concentrations of membrane lipids in the diabetic kidney are altered in response to dapagliflozin treatment. In addition to serving as a selectively permeable barrier that express transmembrane proteins that are sensitive to drug treatment, the plasma membrane is the site where extracellular vesicles (EVs) are released and taken up. 

### 2.4. Dapagliflozin Treatment Decreases EV Release and Excretion into the Urine of db/db Mice during the Inactive Phase

**Figure 4:**
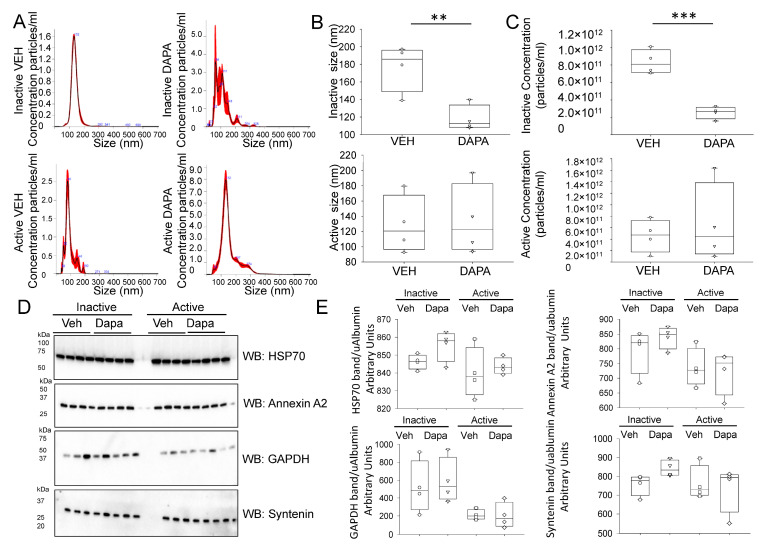
Characterization of uEVs isolated from the inactive and active phases of salt-loaded hypertensive diabetic db/db mice. (**A**) Representative nanoparticle tracking analysis plots of uEVs isolated from diabetic db/db mice treated with vehicle or dapagliflozin during the inactive or active phases. (**B**) Summary plots showing the comparison of uEV size between the two groups and two phases. ** represent a *p*-value of <0.01 (**C**) Summary plots showing the comparison of uEV concentration between the two groups and two phases. *** represent a *p*-value of <0.005 (**D**) Western blots of established uEV markers in each uEV sample from the two groups and phases. n = 4 uEV preparations from inactive-phase urine collections and the same number of uEV preparations from active-phase urine collections of n = 4 vehicle-treated db/db mice and n = 4 dapagliflozin-treated db/db mice. (**E**) Densitometric analysis of the immunoreactive bands in Panel D normalized to urinary albumin levels (uAlbumin).

Urinary extracellular vesicles (uEVs) are a rich source of biomarkers that originate mainly from kidney cell types from each segment of the nephron. In addition, these EVs resemble the cell types that they originate from in the kidney. In order to investigate whether dapagliflozin treatment alters EV size and release in a time-of-day-dependent manner, we isolated EVs from the urine collected during the inactive phase or active phase of vehicle- or dapagliflozin-treated diabetic db/db mice. As shown in Figure 4, the size and concentration of uEVs isolated from urine collected during the inactive phase of diabetic db/db mice treated with dapagliflozin was less compared to diabetic db/db mice treated with the vehicle. The EVs were also characterized by Western blotting for multiple EV markers. HSP70, annexin A2, GAPDH, and syntenin were all found to be enriched in the uEVs from both the vehicle and dapagliflozin groups from both the animals’ inactive and active phases (Figure 4D). Densitometric analysis was performed after normalizing the Western blots to urinary albumin levels. There was no change in any of the four EV markers between the dapagliflozin- and vehicle-treated groups or between the inactive and active cycles (Figure 4E). The decrease in size and concentration of uEVs after dapagliflozin treatment compared to vehicle treatment led us to investigate whether dapagliflozin treatment could also disrupt the organization of lipid rafts within the plasma membrane of cultured renal epithelial cells.

### 2.5. Dapagliflozin Treatment Disrupts Lipid Rafts in Mouse Proximal Tubule Cells

**Figure 5:**
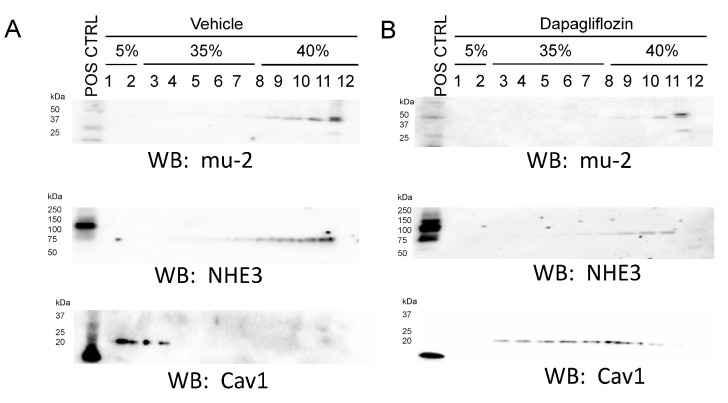
Sucrose density gradient assays from mouse proximal tubule cells treated with dapagliflozin or vehicle. (**A**) Western blot analysis of lipid-raft- and non-lipid-raft-associated fractions from sucrose density gradient fractions of mouse proximal tubule cells treated with vehicle. (**B**) Western blot analysis of lipid-raft- and non-lipid-raft-associated fractions from sucrose density gradient fractions of mouse proximal tubule cells treated with dapagliflozin.

Lipid rafts are signaling platforms enriched in cholesterol and sphingolipids that are involved in diverse cellular processes. In addition to investigating altered plasma membrane lipid composition in diabetic db/db mice, we investigated whether dapagliflozin treatment could disrupt lipid rafts in cultured normal mouse proximal tubule cells. As shown in Figure 5, dapagliflozin treatment in mouse proximal tubule cells resulted in the distribution of the lipid-raft-associated protein caveolin 1 to non-lipid-raft fractions containing the clathrin adaptor protein μ-2 which is primarily localized to high-density sucrose gradient fractions. Also, the amount of sodium hydrogen exchanger (NHE3) protein, which is exclusively expressed at the apical plasma membrane of proximal tubule cells, was reduced in light-density sucrose gradient fractions in dapagliflozin-treated cells compared to cells treated with the vehicle (Figure 5). 

## 3. Discussion

Our study demonstrates for the first time that dapagliflozin treatment alters the concentrations of multiple classes of lipids in the membranes of kidneys from hypertensive diabetic db/db mice. In addition to identifying specific lipids that are affected by dapagliflozin treatment, cellular studies showed dapagliflozin treatment disrupts lipid raft organization in mouse proximal tubule cells. While the efficacy of using dapagliflozin to treat renal and cardiovascular complications in patients with type 2 diabetes has been studied [2], the mechanisms of action are still unknown.

**Figure 6:**
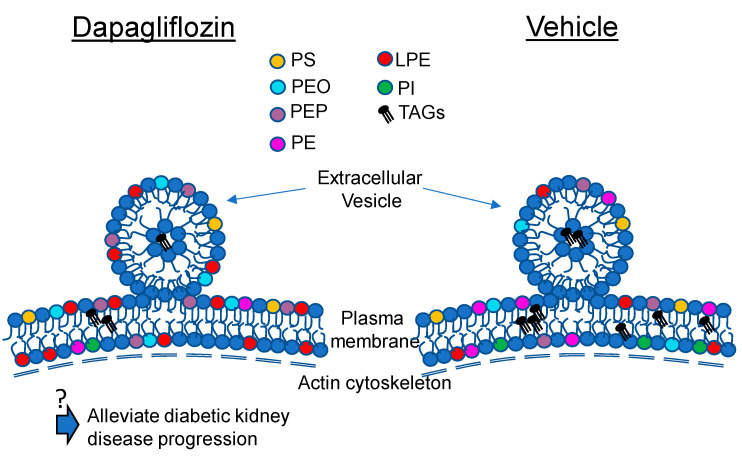
Schematic showing the effects of dapagliflozin treatment on membrane lipids in the kidneys of diabetic db/db mice. An increase in plasmalogen phosphatidylethanolamines in the form of PEPs and PEOs in the membranes of diabetic db/db kidneys after dapagliflozin treatment may alleviate the progression of diabetic kidney disease. ? represents putative effects of dapagliflozin treatment.

In an attempt to address this knowledge gap, we designed a study to investigate changes in the concentration of membrane lipids from hypertensive diabetic db/db mice treated with either dapagliflozin or vehicle. Our lipidomics data identify several bioactive lipids including plasmenyl phosphatidylethanolamines (PEPs) and plasmanyl phosphatidylethanolamines (PE-O) that were increased in the kidney membranes of dapagliflozin-treated mice. The lipidomic data from this study may be relevant to the beneficial effects of dapagliflozin, including reductions in blood glucose and blood pressure. For example, various PEs may play an important role in physiology and pathophysiology. Plasmalogens are phosphatidylcholine or phosphatidylethanolamine glycerophospholipids that have a vinyl ether moiety in the sn-1 position and an esterified fatty acid in the sn-2 position [32]. Sindelar et al. showed plasmalogens effectively protect polyunsaturated fatty acids from oxidative damage [33]. Some of the functions that have been proposed for plasmalogens include modulation of membrane fluidity, mediation of inflammatory responses [34], and anti-oxidative properties [32]. Thus, the increase in plasmalogen phosphatidylethanolamines in the membranes of diabetic db/db kidneys after dapagliflozin treatment (Figure 6) may help to mitigate poor outcomes associated with diabetic kidney disease.

There are numerous strengths of this study. First, we used an established mouse model of type 2 diabetes that develop hypertension after salt loading. Importantly, these db/db mice manifest several characteristics that are often seen in diabetic patients, including obesity and risk of diabetic nephropathy. The diabetic db/db mice used in our study were randomized to a vehicle control group and a dapagliflozin treatment group before being salt-loaded to induce hypertension. In order to minimize bias, we performed a blinded lipidomic study that included using a validated protocol and internal standards.

One limitation of this study is that we did not perform lipidomics using urinary EVs and correlate changes in EV lipids with changes in the lipids in the membranes of kidneys from dapagliflozin-compared to vehicle-treated hypertensive diabetic db/db mice. In general, the lipid compositions of EVs resemble the lipid compositions of the plasma membranes of the parent cells they were released from, and this data may be helpful in identifying the origin of uEVs released in response to dapagliflozin treatment. A second limitation of our study is that we analyzed changes in membrane lipids only from the renal cortex, and we did not investigate regional differences in membrane lipids between the kidney cortex and inner/outer medulla after dapagliflozin treatment. Another limitation of this study is that we were unable to determine whether the decrease in urinary EV concentration observed during the inactive phase of the dapagliflozin-treated diabetic db/db mice compared to the vehicle-treated mice is due to a decreased rate of EV release from different cell types within the nephron or greater uptake of the EVs by recipient cells, thus reducing the amount of EVs excreted into the urine.

Although our study focused on investigating the effects of dapagliflozin in regulating the association between the sodium–hydrogen exchanger (NHE3) and lipid rafts in proximal tubule cells, we realize dapagliflozin may have effects in other segments of the nephron where other membrane transporters and ion channels play a role in diabetic kidney disease and are regulated by lipid rafts. For example, the bumetanide-sensitive sodium–potassium–chloride cotransporter (NKCC2) [35,36] and the amiloride-sensitive epithelial sodium channel (ENaC) [37,38,39] have been found to be associated with lipid rafts. Lipid rafts can mediate endocytosis of membrane proteins, or they can allow for exocytic trafficking of membrane proteins to the cell surface. Further studies are needed to better understand the mechanism by which anti-diabetic drugs such as dapagliflozin regulate epithelial transport mechanisms in the native kidney.

## 4. Materials and Methods

### 4.1. Animals

A total of 16 twelve-week-old, male C57BL/6 db/db mice (BKS.Cg-Dock7m +/+ Leprdb 116 /J; Stock No: 000642) were purchased from the Jackson Laboratory (Bar Harbor, ME, USA). All animal studies were performed under an approved University of Florida Institutional Animal Care and Use Committees protocol and were in compliance with the National Institutes of Health “Guide for the Care and Use of Laboratory Animals”.

### 4.2. Animal Diet

Mice were fed a 4% high-salt diet (HSD) prepared by using type 1 water, agar, NaCl, and powdered chow (Tekland TD.94045.PWD; Envigo; Indianapolis, IN, USA). The diet was dispensed into small food cups and stored at 4 °C for up to 3 days.

### 4.3. Metabolic Cage Studies and Administration of Vehicle or Dapagliflozin

Twelve-week-old male db/db mice were weighted and then placed in individual metabolic cages. These mice were maintained for 10 days on a 4% HSD to induce hypertension. On day 10, the animals’ blood pressure was measured to confirm the development of hypertension (systolic blood pressure greater than 140 mmHg), and the animals were randomly separated into a control group that received a vehicle (saline) and an experimental group that received 1 mg/kg/day dapagliflozin (MedChemExpress, Junction, NJ, USA) for 14 days at 6 p.m. by oral gavage. Urine samples were collected daily between 9 a.m. and 11 a.m. (reflecting urine produced during the night-time active phase) and between 9 p.m. and 11 p.m. (reflecting urine produced during the day-time inactive phase). 

### 4.4. Tissue Lysate Preparation

Fifty milligrams of kidney cortex tissue specimens was washed in 1× of PBS (prepared from the 10× solution (1.37 M NaCl, 27 mM KCl, 100 mM Na2HPO4, 18 mM KH2PO4, PH to 7.4) and then homogenized in 500 µL of tissue protein extraction reagent (TPER; Thermo Fisher Scientific) containing protease and phosphatase inhibitors (Thermo Fisher Scientific, Waltham, MA, USA) using an Omni TH homogenizer (Warrenton, VA, USA). The tissue lysates were centrifuged at 13,000 rpm for 10 min at room temperature before 450 µL of the supernatant was subjected to ultracentrifuging at 34,000 rpm for 30 min at 4 °C. The pellets were resuspended in 200 µL TPER and then sonicated twice for 3 s intervals while on ice. Protein concentrations were determined using a bicinchoninic acid protein assay (BCA) (Thermo Fisher Scientific). 

### 4.5. Lipid Extraction from Kidney Cortex Membrane Fractions

Lipids were extracted using the Bligh and Dyer method [40]. Each sample was adjusted to 1 mL in reagent-grade water and incubated for 10 min on ice. Next, 2 mL of methanol and 0.9 mL methylene chloride were added before being vortexed for 30 s. The EquiSPLASH Lipidomix (Avanti Polar Lipids, Inc., Alabaster, AL, USA) mixture was used as an internal standard, which consists of a mix of 13 deuterated lipids, each at a 100 µg/mL concentration. The deuterated lipids were 15:0–18:1(d7) diacylglycerol (DAG); 18:1(d7) monoacylglycerol (MAG); d18:1–18:1(d9) sphingomyelin (SM); C15 ceramide (CER)-d7; 15:0–18:1(d7)–15:0 triacylglycerol (TAG); 18:1(d7) cholesteryl ester (CE); 15:0–18:1(d7) phosphatidylcholine (PC); 18:1(d7) lysophosphatidylcholine (LPC); 15:0–18:1(d7) phosphatidylglycerol (PG); 15:0–18:1(d7) phosphatidylinositol (PI); 15:0–18:1(d7) phosphatidylethanolamine (PE); 18:1(d7) lysophosphatidylethanolamine (LPE); and 15:0–18:1(d7) phosphatidylserine (PS). The internal standards were diluted 1:5, and 50 μL (20 μg/mL for each standard) was incubated with each sample for 30 min at room temperature. Next, 0.9 mL of methylene chloride and 1 mL of reagent grade water were mixed by inversion 10 times and then centrifuged at 100× *g* for 10 min. The organic phase was collected before a second round of extraction was performed using 2 mL methylene chloride. The pooled organic phases were dried using N2, reconstituted in 50 µL 96% ethanol, and then subjected to liquid chromatography tandem mass spectrometry.

### 4.6. Liquid Chromatography Tandem Mass Spectrometry

Liquid chromatography–electrospray ionization tandem mass spectrometry was performed using an ultra-high-performance liquid chromatography instrument (Shimadzu Co., Kyoto, Japan) and a QTRAP 6500 mass spectrometer (AB SCIEX, Redwood Shores, CA, USA). Lipids were processed and analyzed as previously described by our group [41].

### 4.7. Urinary Extracellular Vesicle Isolation 

Urinary extracellular vesicles were isolated from 10 mL of 12 h urine collections from 8 diabetic db/db mice treated with dapagliflozin or 8 diabetic db/db mice treated with the vehicle. Briefly, the crude urine sample was centrifuged at 1000× *g* at 4 °C for 15 min. Next, 0.22 µm rapid-flow Nalgene filters (Thermo Fisher Scientific) were used to filter the urine samples. The filtered urine samples were subjected to ultracentrifugation at 52,000 rpm at 4 °C for 90 min. The EV pellet was resuspended in 200 µL ultra-pure 1× phosphate-buffered saline (PBS) (Thermo Fisher) and stored at −80 °C. 

### 4.8. Nanoparticle Tracking Analysis

The size and concentration of uEVs were measured using a NanoSight machine as previously described by our group [42].

### 4.9. Urinary Albumin Measurements 

Urine albumin was measured using an albumin assay kit (Catalog #: K551; BioVision/abcam) (Waltham, MA, USA) according to the manufacturer’s instructions.

### 4.10. Western Blot Analysis

Twenty micrograms of total protein were resolved on 4–20% Tris HCl polyacrylamide gels using a Criterion electrophoresis system (BioRad; Hercules, CA, USA) at 200 V for 50 min at room temperature. The separated proteins were subjected to transfer onto nitrocellulose membranes (Thermo Fisher Scientific) by using a Criterion transfer system (Bio-Rad). The membranes were blocked in 5% nonfat dry milk 1× Tris-buffered saline (TBS) (Bio-Rad) (*w*/*v*) for 1 h at room temperature before being incubated in a 1:1000 dilution of primary antibody (anti-HSP70 (ab181606; Abcam, Waltham, MA, USA, annexin A2 (8235; Cell Signaling Tech, Danvers, MA, USA), GAPDH (649203; Biolegend, San Diego, CA, USA), syntenin (ab19903; Abcam) while rocking overnight at 4 °C. Thereafter, the membranes were washed three times with 1× TBS for 5 min intervals and then incubated with horseradish peroxidase-conjugated goat anti-rabbit secondary antibody at a dilution of 1:3000 prepared in blocking solution while on a rocker for 1 h at room temperature. The membranes were then washed four times with 1× TBS for 4 min intervals, incubated with ECL reagent (BioRad) for 7 min at room temperature, and then developed (Bio-Rad imager).

### 4.11. Culture of Mouse Proximal Tubule Cells and Lipid Raft Isolation

Mouse proximal tubule (TKPTS) cells (CRL-3361; ATCC; Manassas, VA, USA) were cultured in DMEM/F12 (50/50) medium with L-glutamine, 7.55 fetal bovine serum, 1× penicillin, and 1× streptomycin and maintained in a humidified incubator with 5% CO_2_ at 37 °C. The medium was changed every 3 days, and the cells were grown to 70% confluence before being treated with the vehicle (medium) or dapagliflozin (5 μM) for 12 h at 37 °C. The cells were scraped in ice-cold 1% Triton X-100/TNEV buffer (10 mM Tris HCl, pH 7.5, 5 mM EDTA, 150 mM NaCl, 2 mM sodium vanadate). The lysates were passed 15 times through a 25-gauge needle and a 1 mL syringe before being incubated on ice for 30 min. The lysates were then subject to centrifugation at 10,000 rpm for 5 min at 4 °C. Next, 500 μL of the supernatants was mixed with 500 μL of 80% sucrose solution in TNE buffer (without Triton X-100). Next, 1800 μL of 35% sucrose in the same buffer was carefully layered on top of the previous layer. Finally, 500 μL of 5% sucrose in the same buffer was carefully layered on top of the 35% layer. The sucrose gradient was then subjected to ultracentrifugation using an SW 50.1 rotor (Beckman) at 34,000 rpm (110,000× *g*) for 22 h at 4 °C. Thereafter, eleven 300 μL fractions were carefully taken from the top to the bottom of the gradient and aliquoted into separate tubes for Western blot analysis. 

### 4.12. Statistical Analysis

A Student *t*-test was used to determine statistical significance between two groups, while a one-way ANOVA was used to determine statistical significance for more than two groups. The SigmaPlot software (Systat Software, San Jose, CA, USA) was used for all statistical analyses. The values presented are expressed as mean ± SE, and a *p*-value of less than 0.05 was considered statistically significant.

## 5. Conclusions

Taken together, these results suggest specific membrane lipids in the diabetic kidney and lipid raft organization in proximal tubule cells are sensitive to dapagliflozin treatment. Accordingly, the bioactive lipids in diabetic kidney membranes that were attenuated or augmented after dapagliflozin treatment may have the potential to be used as biomarkers and help to elucidate key mechanisms associated with the beneficial effects of dapagliflozin in diabetic kidney disease.

## Data Availability

The data are included within the figures of this manuscript.

## References

[1] Ferrannini E. (2017). Sodium-Glucose Co-transporters and Their Inhibition: Clinical Physiology. Cell Metab..

[2] Fioretto P., Zambon A., Rossato M., Busetto L., Vettor R. (2016). SGLT2 Inhibitors and the Diabetic Kidney. Diabetes Care.

[3] Wiviott S.D., Raz I., Sabatine M.S. (2019). Dapagliflozin and Cardiovascular Outcomes in Type 2 Diabetes. N. Engl. J. Med..

[4] Ishizawa K., Wang Q., Li J., Xu N., Nemoto Y., Morimoto C., Fujii W., Tamura Y., Fujigaki Y., Tsukamoto K. (2019). Inhibition of Sodium Glucose Cotransporter 2 Attenuates the Dysregulation of Kelch-Like 3 and NaCl Cotransporter in Obese Diabetic Mice. J. Am. Soc. Nephrol..

[5] Varzideh F., Kansakar U., Santulli G. (2021). SGLT2 inhibitors in cardiovascular medicine. Eur. Hear. J.-Cardiovasc. Pharmacother..

[6] Bellido V., Martínez J., Calvo F., Villarroel A., Lecumberri E., Moreno J., Morillas C., Rodrigo S., Izarra A., Lecube A. (2022). Beyond the Glycaemic Control of Dapagliflozin: Microangiopathy and Non-classical Complications. Diabetes Ther..

[7] Euh W., Lim S., Kim J.-W. (2021). Sodium-Glucose Cotransporter-2 Inhibitors Ameliorate Liver Enzyme Abnormalities in Korean Patients With Type 2 Diabetes Mellitus and Nonalcoholic Fatty Liver Disease. Front. Endocrinol..

[8] Ma C., De Baaij J.H., Millar P.J., Gault V.A., De Galan B.E., Bindels R.J., Hoenderop J.G. (2019). Effect of Dapagliflozin Treatment on the Expression of Renal Sodium Transporters/Channels on High-Fat Diet Diabetic Mice. Nephron.

[9] Park S.-H., Belcastro E., Hasan H., Matsushita K., Marchandot B., Abbas M., Toti F., Auger C., Jesel L., Ohlmann P. (2021). Angiotensin II-induced upregulation of SGLT1 and 2 contributes to human microparticle-stimulated endothelial senescence and dysfunction: Protective effect of gliflozins. Cardiovasc. Diabetol..

[10] Seijas M.C., Agra-Bermejo R.M., Fernández A.L., Martínez-Cereijo J.M., Sierra J., Soto-Pérez M., Rozados-Luis A., Juanatey J.R.G., Eiras S. (2019). High released lactate by epicardial fat from coronary artery disease patients is reduced by dapagliflozin treatment. Atherosclerosis.

[11] Al-Sharea A., Murphy A.J., Huggins L., Hu Y., Goldberg I.J., Nagareddy P.R. (2018). SGLT2 inhibition reduces atherosclerosis by enhancing lipoprotein clearance in Ldlr type 1 diabetic mice. Atherosclerosis.

[12] Rau M., Thiele K., Hartmann N.-U.K., Möllmann J., Wied S., Böhm M., Scharnagl H., März W., Marx N., Lehrke M. (2021). Effects of empagliflozin on lipoprotein subfractions in patients with type 2 diabetes–Data from a randomized, placebo-controlled study. Atherosclerosis.

[13] Lv Q., Le L., Xiang J., Jiang B., Chen S., Xiao P. (2020). Liver Transcriptomic Reveals Novel Pathways of Empagliflozin Associated With Type 2 Diabetic Rats. Front. Endocrinol..

[14] Mone P., Varzideh F., Jankauskas S.S., Pansini A., Lombardi A., Frullone S., Santulli G. (2022). SGLT2 Inhibition via Empagliflozin Improves Endothelial Function and Reduces Mitochondrial Oxidative Stress: Insights From Frail Hypertensive and Diabetic Patients. Hypertension.

[15] Chen L., LaRocque L.M., Efe O., Wang J., Sands J.M., Klein J.D. (2016). Effect of Dapagliflozin Treatment on Fluid and Electrolyte Balance in Diabetic Rats. Am. J. Med. Sci..

[16] Saeedi P., Petersohn I., Salpea P., Malanda B., Karuranga S., Unwin N., Colagiuri S., Guariguata L., Motala A.A., Ogurtsova K. (2019). Global and regional diabetes prevalence estimates for 2019 and projections for 2030 and 2045: Results from the International Diabetes Federation Diabetes Atlas, 9th edition. Diabetes Res. Clin. Pract..

[17] Paneni F., Beckman J., Creager M.A., Cosentino F. (2013). Diabetes and vascular disease: Pathophysiology, clinical consequences, and medical therapy: Part I. Eur. Hear. J..

[18] Fryar C.D., Ostchega Y., Hales C., Zhang G., Kruszon-Moran D. (2017). Hypertension Prevalence and Control Among Adults: United States, 2015-2016. NCHS Data Brief..

[19] Filippone E.J., Ruzieh M., Foy A. (2020). Thiazide-Associated Hyponatremia: Clinical Manifestations and Pathophysiology. Am. J. Kidney Dis..

[20] Richards J., Gumz M.L. (2013). Mechanism of the circadian clock in physiology. Am. J. Physiol. Integr. Comp. Physiol..

[21] van Balkom B.W., Pisitkun T., Verhaar M.C., Knepper M.A. (2011). Exosomes and the kidney: Prospects for diagnosis and therapy of renal diseases. Kidney Int..

[22] Miranda K.C., Bond D.T., McKee M., Skog J., Păunescu T.G., Da Silva N., Brown D., Russo L.M. (2010). Nucleic acids within urinary exosomes/microvesicles are potential biomarkers for renal disease. Kidney Int..

[23] Jella K.K., Yu L., Yue Q., Friedman D., Duke B.J., Alli A.A. (2016). Exosomal GAPDH from Proximal Tubule Cells Regulate ENaC Activity. PLoS ONE.

[24] Shi Z., Wang Q., Zhang Y., Jiang D. (2020). Extracellular vesicles produced by bone marrow mesenchymal stem cells attenuate renal fibrosis, in part by inhibiting the RhoA/ROCK pathway, in a UUO rat model. Stem Cell Res. Ther..

[25] Zhao P., Zhu Y., Sun L., Zhu W., Lu Y., Zhang J., Mao Y., Chen Q., Zhang F. (2021). Circulating Exosomal miR-1-3p from Rats with Myocardial Infarction Plays a Protective Effect on Contrast-Induced Nephropathy via Targeting ATG13 and activating the AKT Signaling Pathway. Int. J. Biol. Sci..

[26] Zhu M., Sun X., Qi X., Xia L., Wu Y. (2020). Exosomes from high glucose-treated macrophages activate macrophages and induce inflammatory responses via NF-κB signaling pathway in vitro and in vivo. Int. Immunopharmacol..

[27] Pekkucuksen N.T., Liu L.P., Aly R., Shoemaker L.R., Alli A.A. (2022). Extracellular vesicles from focal segmental glomerulosclerosis pediatric patients induce STAT3 activation and mesangial cell proliferation. PLoS ONE.

[28] Hirohama D., Nishimoto M., Ayuzawa N., Kawarazaki W., Fujii W., Oba S., Shibata S., Marumo T., Fujita T. (2021). Activation of Rac1-Mineralocorticoid Receptor Pathway Contributes to Renal Injury in Salt-Loaded *db/db* Mice. Hypertension.

[29] Sas K.M., Lin J., Wang C.-H., Zhang H., Saha J., Rajendiran T.M., Soni T., Nair V., Eichinger F., Kretzler M. (2021). Renin-angiotensin system inhibition reverses the altered triacylglycerol metabolic network in diabetic kidney disease. Metabolomics.

[30] Yoshioka K., Hirakawa Y., Kurano M., Ube Y., Ono Y., Kojima K., Iwama T., Kano K., Hasegawa S., Inoue T. (2021). Lysophosphatidylcholine mediates fast decline in kidney function in diabetic kidney disease. Kidney Int..

[31] Wiggenhauser L.M., Metzger L., Bennewitz K., Soleymani S., Boger M., Tabler C.T., Hausser I., Sticht C., Wohlfart P., Volk N. (2022). *pdx1* Knockout Leads to a Diabetic Nephropathy– Like Phenotype in Zebrafish and Identifies Phosphatidylethanolamine as Metabolite Promoting Early Diabetic Kidney Damage. Diabetes.

[32] Wallner S., Schmitz G. (2011). Plasmalogens the neglected regulatory and scavenging lipid species. Chem. Phys. Lipids.

[33] Sindelar P.J., Guan Z., Dallner G., Ernster L. (1999). The protective role of plasmalogens in iron-induced lipid peroxidation. Free. Radic. Biol. Med..

[34] Bozelli J.C.J., Azher S., Epand R.M. (2021). Plasmalogens and Chronic Inflammatory Diseases. Front. Physiol..

[35] Ares G.R., Ortiz P.A. (2012). Dynamin2, Clathrin, and Lipid Rafts Mediate Endocytosis of the Apical Na/K/2Cl Cotransporter NKCC2 in Thick Ascending Limbs. J. Biol. Chem..

[36] Welker P., Böhlick A., Mutig K., Salanova M., Kahl T., Schlüter H., Blottner D., Ponce-Coria J., Gamba G., Bachmann S. (2008). Renal Na^+^-K^+^-Cl^−^cotransporter activity and vasopressin-induced trafficking are lipid raft-dependent. Am. J. Physiol. Physiol..

[37] Hill W.G., Butterworth M.B., Wang H., Edinger R.S., Lebowitz J., Peters K.W., Frizzell R.A., Johnson J.P. (2007). The Epithelial Sodium Channel (ENaC) Traffics to Apical Membrane in Lipid Rafts in Mouse Cortical Collecting Duct Cells. J. Biol. Chem..

[38] Lee I.-H., Campbell C., Song S.-H., Day M.L., Kumar S., Cook D.I., Dinudom A. (2009). The Activity of the Epithelial Sodium Channels Is Regulated by Caveolin-1 via a Nedd4-2-dependent Mechanism. J. Biol. Chem..

[39] Tuna K.M., Liu B.-C., Yue Q., Ghazi Z.M., Ma H.-P., Eaton U.C., Alli A.A. (2019). Mal protein stabilizes luminal membrane PLC-β3 and negatively regulates ENaC in mouse cortical collecting duct cells. Am. J. Physiol. Physiol..

[40] Bligh E.G., Dyer W.J. (1959). A rapid method of total lipid extraction and purification. Can. J. Biochem. Physiol..

[41] Glover S.C., Nouri M., Tuna K.M., Alvarez L.B.M., Ryan L.K., Shirley J.F., Tang Y., Denslow N.D., Alli A.A. (2019). Lipidomic analysis of urinary exosomes from hereditary α-tryptasemia patients and healthy volunteers. FASEB BioAdv..

[42] Chacko K.M., Nouri M.-Z., Schramm W.C., Malik Z., Liu L.P., Denslow N.D., Alli A.A. (2021). Tempol Alters Urinary Extracellular Vesicle Lipid Content and Release While Reducing Blood Pressure during the Development of Salt-Sensitive Hypertension. Biomolecules.

